# Photonics Breakthroughs 2025: Mask-Based Integrated Miniscope for Large-scale Volumetric Imaging

**DOI:** 10.1109/jphot.2026.3706745

**Published:** 2026-06-23

**Authors:** Feng Tian, Weijian Yang

**Affiliations:** The authors are with the Department of Electrical and Computer Engineering, University of California, Davis, CA 95616 USA

**Keywords:** 3D imaging, calcium imaging, computational imaging, head-mounted, integrated imaging, mask-based imaging, lensless imaging, miniaturized microscope, miniscope, volumetric imaging

## Abstract

Mask-based integrated microscopy represents a powerful computational imaging paradigm, enabling 3D imaging over a large field of view within a thin device footprint. This architecture leverages a thin optical mask to encode object information onto a camera sensor placed in close proximity. A major challenge in this field is to maintain high reconstruction quality amid low signal-to-background ratios, while simultaneously reducing computational requirements to allow for larger reconstruction volumes. Here, we describe a significant breakthrough in this area: DeepInMiniscope, a system featuring a highly scalable reconstruction algorithm for high-quality imaging over large 3D volumes. We detail the joint development of the hardware and reconstruction algorithms that enable this high-quality, efficient, and robust imaging framework, and present its capability to record neural activity in the mouse brain with near-cellular resolution. DeepInMiniscope represents a key step towards large-scale, 3D functional recording in freely-behaving animals.

## I. Introduction

### A. Background

Fluorescence microscopy is a fundamental imaging technique that enables visualization of biological structures and dynamics with high molecular specificity by labeling targets with fluorescent markers. It plays a central role in biological and biomedical research, supporting studies of cellular dynamics, physiological signaling, and pathological pathways across multiple spatial and temporal scales. While traditional fluorescence microscopes offer high image quality via lens-based optics, they are often constrained by bulky footprints and inherent tradeoffs between resolution, field of view (FOV), and system portability. Such portability is particularly critical for endoscopic and implantable applications. For instance, in the context of in vivo brain imaging in freely moving animals, compact systems offer a unique opportunity to study biological processes under natural conditions. Over the past years, significant efforts have been devoted to miniaturize the optics to reduce the footprint of microscopes [1], [2], [3], [4], [5], [6], [7], [8], [9]. However, because the underlying architecture remains largely unchanged, the same fundamental tradeoffs persist. Furthermore, like their benchtop counterparts, these systems typically image only one axial plane at a time and suffer from substantial background noise from out-of-focus light. There is a growing demand for systems that can simultaneously achieve a wide FOV, volumetric imaging capability, and high spatiotemporal resolution. Simultaneously realizing these features remains a central challenge in modern microscopy design.

Computational imaging has emerged as a powerful paradigm that integrates the design of optical hardware and reconstruction algorithms to overcome the physical limitations of traditional systems [10]. A notable example is light-field microscopy [11], [12], which enables single-shot 3D imaging by incorporating a microlens array into imaging system. Among various strategies, mask-based integrated imaging [13], [14], [15], [16], [17], [18], [19], [20], [21], [22], sometimes referred as lensless imaging, offers a compelling solution for single-shot 3D acquisition with a scalable FOV while maintaining spatial resolution without increasing device thickness. In this architecture, a thin optical mask replaces bulky lenses as the primary image encoder. For microscopic applications, the object is positioned in close proximity to the mask, which is itself situated near the sensor, resulting in an exceptionally compact footprint. In this configuration, distinct regions of the object are projected through different portions of the mask onto the camera sensor. Consequently, the FOV scales directly with the mask and sensor dimensions, independent of device thickness. By employing multiplexing strategies, the mask encodes high-dimensional information that is subsequently recovered through computational decoding. A classic example is a microlens array mask, where each object point is captured by multiple lenses [16], [17], [18], [19], [20]. In this setup, the point spread function (PSF) varies according to object depth, allowing 3D volumetric data to be reconstructed from a single 2D measurement.

### B. Challenges

As a computational paradigm, mask-based integrated microscopy depends fundamentally on reconstruction algorithms to retrieve object information. The primary challenges facing this approach center on the quality, efficiency and robustness of the reconstruction process. First, masks used in these systems, such as microlens arrays [16], [17], [18], [19], [20] or diffusers [14], typically exhibit a spatially dispersed PSF. Because a single object point is encoded across many pixels, overlapping signals from non-sparse objects significantly increase the background level. Such overlap is generally more severe than in light-field microscopy, as it is typically global, in contrast to the more localized and geometrically structured spreading characteristic of light-field systems. This issue is particularly severe in in vivo fluorescence imaging, where inherently weak signals lead to a low signal-to-background ratio (SBR), resulting in a highly challenging ill-posed inverse problem. Second, conventional iterative optimization algorithms, such as the Alternating Direction Method of Multipliers (ADMM) or Richardson-Lucy (RL) deconvolution, rely on an accurate forward model. In mask-based systems where the PSF is spatially variant, the corresponding system matrix becomes massive. This imposes a heavy computational overhead and high memory requirements, which limit scalability for high-resolution 3D reconstruction. Furthermore, the iterative nature of these solvers often results in slow processing speeds. Finally, while deep neural networks can accelerate reconstruction and mitigate noise, they often require vast amounts of high-quality training data. This introduces a robustness concern: the reconstruction quality becomes highly dependent on the training distribution. Such models often lack generalizability, and fail to produce reliable results when applied to sample types or imaging conditions that differ from the training dataset. This could limit the practical biological applications.

### C. Breakthroughs

We developed DeepInMiniscope [23], a mask-based integrated miniaturized microscope system that leverages a custom-designed optical mask alongside physics-informed computational algorithms to significantly enhance reconstruction quality, efficiency, and robustness. To overcome the scalability issues inherent in traditional computational microscopy, we introduced the concept of the local PSF. By ensuring that each object point contributes only to a localized region on the sensor rather than spanning the entire FOV, we enabled the decomposition of the reconstruction process into overlapping, parallelizable local blocks. This was achieved through a doublet lens design, which maintains a high Strehl ratio within the effective imaging area while effectively suppressing non-local signal contributions. These optical strategies provide a scalable solution for high-dimensional information capture, and support high multiplexing with controlled signal spread.

Building upon this hardware foundation, we developed two distinct reconstruction frameworks to address the bottlenecks of conventional solvers. The first algorithm, multi-local-FOV ADMM-Net [23], is an unrolled neural network that mimics the ADMM architecture using learnable parameters. This approach integrates the rigorous physics of iterative optimization with the speed and denoising capabilities of deep learning. Crucially, by embedding the imaging physics without requiring large-scale matrix multiplication, it enhances reconstruction robustness while bypassing high computational costs. Furthermore, the local reconstruction framework facilitates FOV-level parallelization, enabling fast reconstruction via multi-GPU processing. In addition to neural network, we also develop a second reconstruction method, which is a list-based Richardson-Lucy (RL) algorithm [23] that indexes only sparse, meaningful voxel-pixel connections. By focusing exclusively on these significant connections, the framework avoids the storage and processing of massive, redundant system matrices, and leads to substantial memory savings compared to standard iterative optimization methods.

We validated the performance of DeepInMiniscope across a diverse range of challenging biological specimens. We demonstrated high-quality, high-speed imaging of scattering phantoms with complex, dense features, as well as dynamic organisms such as Caenorhabditis elegans (C. elegans) and hydra exhibiting rapid 3D motion. Most notably, we successfully recorded cortical neural activity at near-cellular resolution in awake mice, underscoring the system’s potential for high-resolution in vivo brain imaging under natural conditions.

## II. Breakthrough Research

### A. DeepInMiniscope: A Physics-Informed Computational Framework for Integrated Miniscope

#### 1) Concept and Principle:

**Fig. 1.**
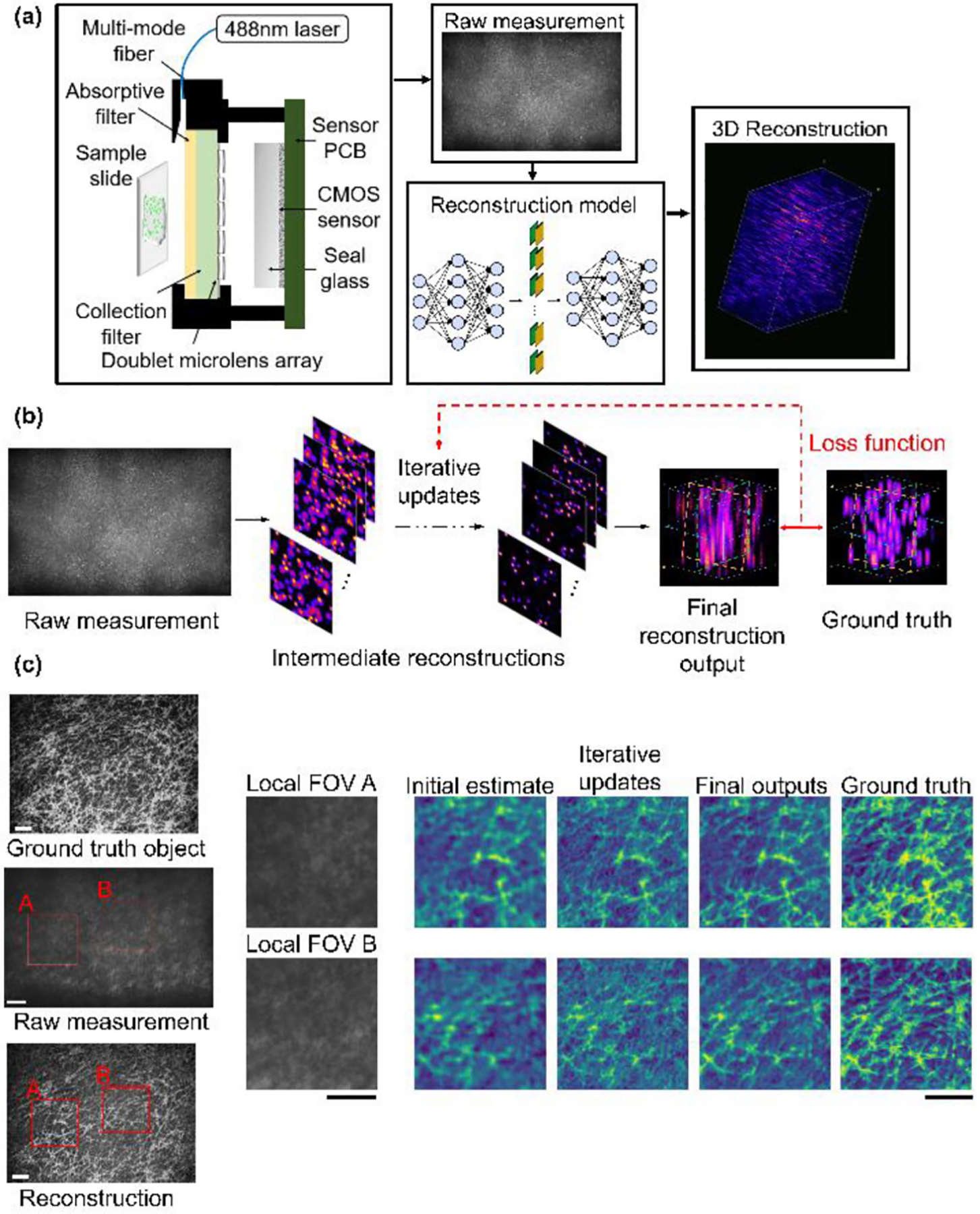
Overview of DeepInMiniscope and the reconstruction algorithm. (a) Schematic of DeepInMiniscope. The raw measurement captured by the miniscope is processed through a physics-informed deep network to reconstruct the objects in 3D. (b) Iterative 3D reconstruction. The volumetric object is iteratively recovered and refined from the input measurement using a learned optimization approach. (c) Localized reconstruction framework. The raw measurement is partitioned into overlapping local FOVs, each of which is independently processed by an ADMM-Net. The final high-resolution 3D reconstruction is then fused from these distributed local outputs, enabling scalable processing of large-scale volumes. Scale bar: 500 *μ*m. Figure reprinted and adopted from [23].

**Fig. 2.**
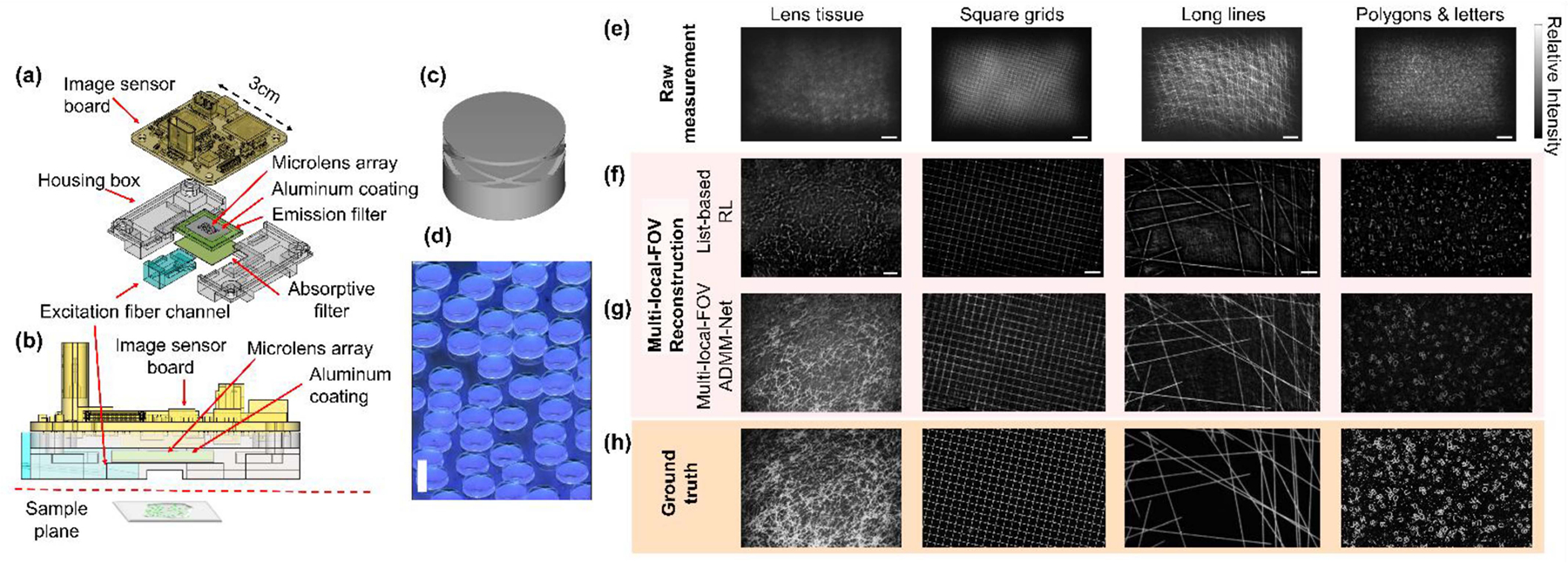
Assembly of DeepInMiniscope and validation on 2D fluorescent samples. (a) Exploded view of DeepInMiniscope, illustrating the 3D-printed housing, the optical filter stack with the integrated microlens array, and the CMOS sensor. (b) Side view of the fully assembled miniscope. (c) Structure of the double lens unit. (d) Micrograph of the fabricated microlens array. (e)–(h) Performance validation on 2D fluorescent samples. (e) Raw measurements. (f) Reconstruction using list-based RL algorithm. (g) Reconstruction using a multi-local-FOV ADMM-Net. (h) Ground truth image acquired with a high-resolution benchtop microscope. Scale bar, 500 *μ*m. Figure reprinted and adopted from [23].

The DeepInMiniscope architecture comprises a custom-designed microlens array integrated directly onto an emission filter in close proximity to the image sensor (Figs. 1(a), 2(a)–(d)). Unlike stochastic masks such as diffusers, the microlens array produces a structured, sparse PSF that inherently minimizes background interference. Each microlens unit has an effective imaging FOV. We optimized these units as doublets to sharpen the PSF within the effective FOV while suppressing signal contributions from adjacent regions. This optical confinement is a critical prerequisite for the scalable reconstruction algorithms described hereafter. Furthermore, the microlens array follows a structured yet pseudo-uniform pattern to ensure consistent angular sampling across the entire FOV, providing stable 3D multiplexing and uniform reconstruction performance.

The reconstruction framework of DeepInMiniscope distinguishes itself through its ability to handle spatially variant PSFs while maintaining computational scalability. We developed a multi-local-FOV ADMM-Net that partitions the global FOV into multiple overlapping local-FOVs, which are reconstructed individually (Fig. 1(b)–(c)). Each local-FOV is processed via an unrolled neural network that mimics the ADMM framework, where each stage represents a single iteration with learnable parameters. The resulting local reconstructions are subsequently fused; this overlapping fusion stabilizes voxel estimation by providing multiple spatial references, thereby mitigating the impact of individual lens unit variability. Our multi-local-FOV ADMM-Net is characterized by three distinct features. First, it utilizes local FOV reconstruction. Compared to a monolithic global reconstruction, our approach circumvents the complexities of global spatial variance, as the PSF can be accurately approximated as shift-invariant within the local-FOV. This strategy also significantly reduces GPU RAM requirements by avoiding large-scale 3D volume processing. Second, the architecture is computationally optimized for high efficiency. Unlike standard ADMM-Nets that rely on intensive matrix multiplications, our architecture leverages PSF sparsity to convert these operations into Hadamard products in the Fourier domain, which drastically reduces computational overhead. Third, the neural network is physics-informed. By embedding the physical model of image formation directly into the network and utilizing a Fourier-domain Hadamard network for deconvolution, we ensure stable convergence and the recovery of sharp spatial features. To maintain high fidelity, we implemented a closed-loop loss function to enforce consistency between the reconstructed objects and reprojected measurements, alongside a multi-view 3D projection loss to prevent degenerate solutions and tighten axial confinement.

In addition to multi-local-FOV ADMM-Net, we developed a list-based RL algorithm specifically optimized for spatially variant, sparse PSFs. Conventional RL deconvolution typically necessitates a massive system matrix to store spatially dependent PSFs, which could pose a challenge on hardware memory. By exploiting the sparsity of our optical encoding, the list-based RL algorithm stores only the explicitly strong voxel-pixel connections. This “list-based” indexing strategy bypasses the storage and processing of redundant, non-informative data. It results in substantial memory savings compared to standard iterative optimization methods and enables high-resolution 3D reconstruction.

#### 2) Results and Achievements:

The DeepInMiniscope features a wide FOV of 4 mm by 6 mm. The working distance between the object and the microlens array is ~3.9 to 5.5 mm, and the gap between the microlens array and the image sensor is ~1.6 mm. Excitation light is delivered via two integrated optical fibers, and the resulting fluorescence is captured through the doublet microlens array on a filter stack. DeepInMiniscope achieves a lateral resolution of ~10 *μ*m and an axial resolution of ~60 *μ*m. The entire assembly is exceptionally compact, measuring 30 mm by 30 mm by 6.5 mm and weighing only 7.5 g (reduced to 3 g if excluding the camera).

Our highly efficient reconstruction algorithms enable the recovery of large-scale 3D objects that were previously computationally challenging. Using the multi-local-FOV ADMM-Net, we achieved 3D reconstructions over a 6 mm by 4 mm by 0.6 mm volume (corresponding to 1248 × 832 × 13 voxels) at frame rates of 1 Hz using 24 GB of GPU RAM, or 3 Hz with 80 GB of GPU RAM. This represents a substantial leap over conventional ADMM: while standard ADMM can theoretically provide high-quality reconstructions, the memory requirement for a data volume of this scale (N*~*10^6^–10^7^ voxels) would reach tens of terabytes (TB), rendering it practically unfeasible for standard laboratory hardware.

For the list-based RL algorithm, we exploit the fact that each object voxel maps to fewer than 50 camera pixels. This sparsity effectively reduces the complexity of the system matrix from *O*(*N*^2^) to *O*(50*N*), which significantly conserves memory and enables processing on a standard laptop. The list-based RL runs at 0.58 to 12 s per iteration for approximately 1.1 × 10^7^ voxels across 13 to 16 planes, and it typically requires only three to five iterations for convergence on a typical laptop. While list-based RL may be less suited than ADMM-Net for reconstructing complex, continuous features, it offers the distinct advantage of being training-free and providing greater flexibility across diverse sample types.

**Fig. 3.**
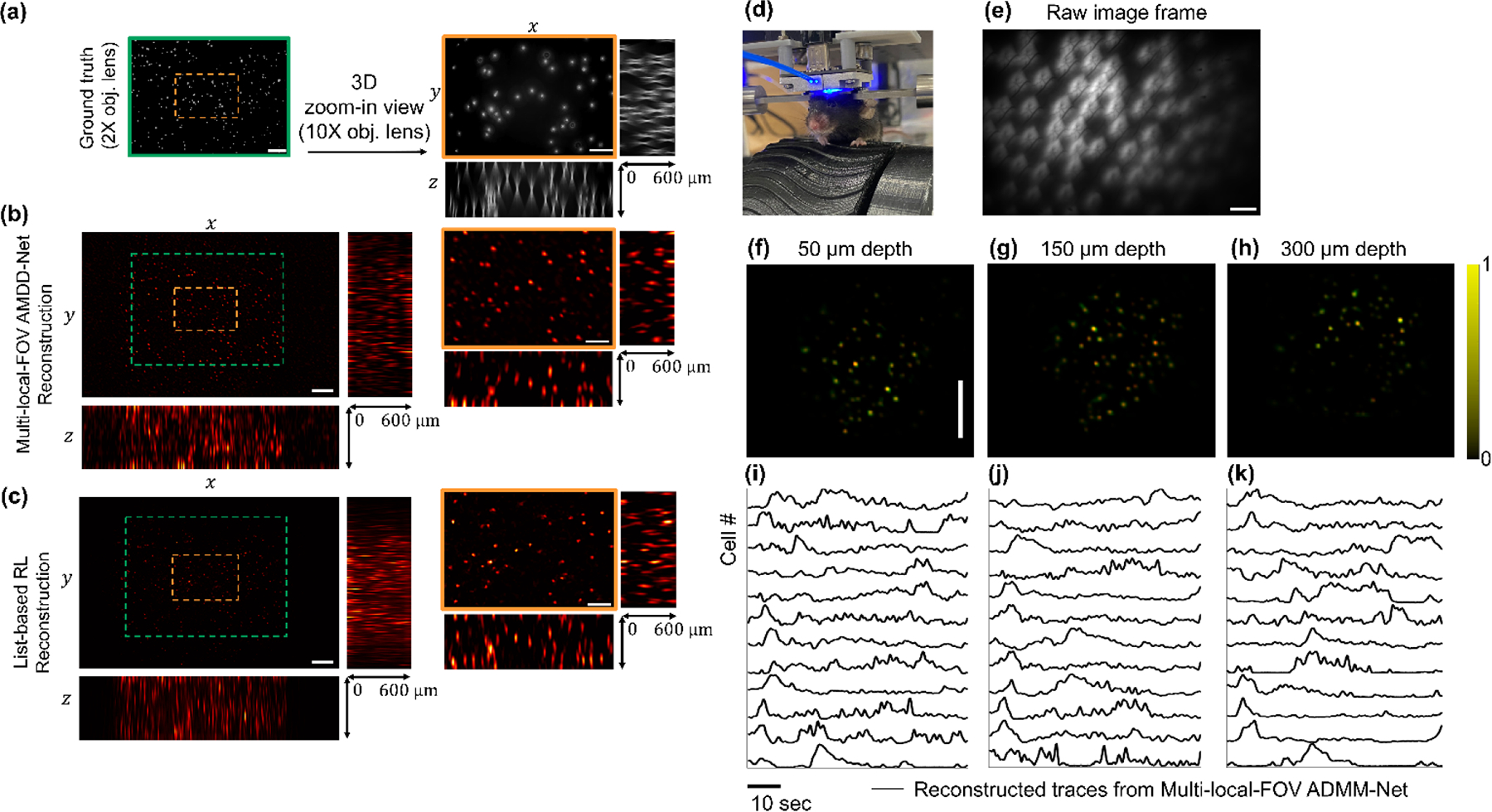
3D volumetric imaging of fluorescent phantoms and in vivo neural activity. (a-c) Volumetric imaging of fluorescent beads. (a) Fluorescent bead phantom (5μm diameter) distributed in an optical clear polymer, imaged by a benchtop microscope with 2× objective lens (left, xy view) and 10× objective lens (right, 3D views). (b) 3D reconstruction results across a 600 *μ*m depth range through the multi-local-FOV ADMM-Net. The green and orange dashed boxes in the xy view correspond to the respective 2× and 10× objective lens fields in (a). Right: Zoom-in view of the region within the orange dashed box. (c) Comparative reconstruction of the same volume using the list-based RL algorithm. (d-k) In vivo calcium imaging of neural activity in the mouse visual cortex transfected with GCaMP6f. (d) Experimental setup. (e) A representative raw measurement frame. (f-h) Reconstructed planes at three distinct depths, displayed as spatiotemporal correlation map of the neural activity. The individual neurons were indicated by red color. (i-k) Representative normalized temporal traces showing the activity of the neurons extracted from the planes in (f-h). Scale bars, 500 *μ*m [(a-c), left], 200 *μ*m [(a-c), right], 500 *μ*m (e) and (f). Figure reprinted and adopted from [23].

DeepInMiniscope has demonstrated high-quality imaging across a wide range of challenging biological and synthetic specimens (Figs. 2(e)–(h), 3). The system successfully resolved randomly distributed 5 *μ*m fluorescence beads over a 6 mm by 4 mm by 0.6 mm volume across 13 axial planes. Notably, it maintained robust 3D reconstruction of 12 *μ*m beads embedded in non-fluorescent scattering media across mean free paths ranging from 50 to 500 *μ*m. In biological applications, the microscope resolved C. elegans embryos as small as 13 *μ*m and captured the dynamic 3D motion of live hydra over a 6 mm by 4 mm FOV with a 2-mm depth range. Finally, the system facilitated in vivo imaging of neuronal activity in the awake mouse cortex with near-cellular resolution (*~*100 *μ*m axial FWHM). This cortical recording spanned a 3D volume of approximately 1.5 mm by 2 mm by 300 *μ*m, and successfully extracted the neural dynamics across multiple planes at depths of 50, 150, and 300 *μ*m.

### B. Recent Advancements in Mask-Based Integrated Imaging and Computational Reconstruction Algorithms

#### 1) Fourier-domain Learning Frameworks:

In DeepInMiniscope, a Fourier-domain Hadamard network is employed for deconvolution, where a frequency-domain kernel related to the optical transfer function (OTF) is learned. This approach is particularly well-suited for spatially sharp PSFs characterized by broad spectral support in the OTF. Compared to spatial-domain convolution, Hadamard multiplication in the Fourier domain offers superior computational efficiency [23]. This strategy has been increasingly adopted in recent literature. Within the context of mask-based integrated miniscopes, SV-FourierNet [24] was introduced for object reconstruction, which addresses spatially varying aberrations through a multi-channel Fourier neural network with learned global receptive fields. Fourier-ADMM networks [25] which integrate Fourier-domain inversion with ADMM regularization and backpropagation was recently proposed, enabling fast and accurate reconstruction while preserving the underlying imaging physics. In the realm of extended depth-of-field microscopy, a physics-informed multi-Hadamard-Net was used for 3D reconstruction in a microscope with a V-shaped PSF [26]. The network architecture learns depth-specific deconvolution kernels in the frequency domain across multiple branches before a 3D U-Net fuses the recovered volume. Collectively, these studies demonstrate that Fourier-domain learning has emerged as a practical and scalable solution to encode physically meaningful global deconvolution behavior while maintaining high network efficiency.

#### 2) Unrolled Optimization Models:

DeepInMiniscope uses an unrolled neural network, which integrates the strength of the conventional iterative model-based optimization algorithms and data-driven learning. Each iteration in the model-based optimization is turned into a learnable network, which learns task-specific updates from data. Beyond the multi-local-FOV ADMM-Net used in the DeepInMiniscope, other unrolled neural networks were used in mask-based imagers for object reconstructions, such as the learned ADMM [27] and unrolled primal-dual model [28].

Most recently, building from our list-based RL method, we developed an unrolled RL deconvolution network with partially connected layers (PC-RLN) for mask-based imager [29]. Each stage of the network mimics an iteration of the RL algorithm, and incorporates a learnable, partially connected layer to model both the forward imaging process and the back-projection based on the PSF. Compared with other unrolled neural network, PC-RLN has a simplified and efficient network structure based on the sparse mapping connections between the object voxels and image pixels, and offer high interpretability as the original RL algorithm. It further enables a self-supervised learning scheme where the paired object and measured images training data are not required.

#### 3) Multi-kernel Deconvolution and Coordinate-Conditioned Convolution:

While the multi-local-FOV ADMM-Net and list-based RL in DeepInMiniscope are specifically engineered to manage severe spatially variant PSFs through localized processing and sparse indexing, alternative strategies address these challenges differently. For instance, MultiWienerNet [30] and SV-FourierNet [24] learn a set of global deconvolution kernels to reconstruct the entire scene simultaneously, subsequently blending the results into a final output. Because each learned kernel operates globally, in contrast to the localized sub-fields processed by multi-local-FOV ADMM-Net, this multi-global approach is better suited for imaging systems where the degree of shift variance is relatively mild.

Another approach to handle spatially variant PSF is coordinate-conditioned convolution [31]. In a recent demonstration for mask-based integrated microscopy [32], a lightweight multi-layer perceptron (MLP) was employed to map positional encodings to spatially varying masks. These masks are applied multiplicatively to feature maps to locally adapt reconstruction kernels, facilitating position-aware deconvolution with minimal memory requirements and bypassing the need for global operators.

#### 4) Coordinate-based Neural Network:

While most reconstruction algorithms, including those used in DeepInMiniscope, rely on a discretized, fixed-grid voxel representation, coordinate-based neural networks (also known as implicit neural representation or neural fields) [33], [34]) have emerged as a compelling alternative. Such a method models the object as a continuous function rather than a discrete volume. In a recently reported single-shot volumetric fluorescence microscope, the 3D object was represented as an implicit neural function which maps spatial coordinates directly to intensity values [35]. Compared to classical deconvolution methods such as RL, this paradigm improves reconstruction fidelity and enables large-scale imaging without the constraints of explicit voxel discretization. By treating the object as a continuous entity, these coordinate-based frameworks offer a high degree of flexibility in representing complex biological structures across multiple scales. Furthermore, coordinate-based neural networks have been utilized to model both the object and motion dynamics, serving as a continuous space-time prior to reconstruct videos captured by a lensless imager [36]. This approach improved the reconstruction quality compared to traditional methods that process frames independently in a frame-by-frame manner.

#### 5) Innovation of Mask Design in Integrated Imaging:

While DeepInMiniscope uses a microlens array, recent years have witnessed significant innovations in the mask design for integrated imaging. For instance, a programmable Fresnel zone aperture (FZA) based on liquid crystals or multi-phase FZA framework was employed to enhance imaging resolution and the signal-to-noise ratio [37], [38]. Additionally, diffractive optical elements have been utilized as imaging optics for miniaturized mesoscopes, providing an extended depth of field for recording neuronal activity in freely behaving mice [39]. Other developments include metalens arrays designed to achieve a large FOV in miniaturized microscopy [40] and multilayer masks used to optimize the condition number of the optical system’s transmission matrix, thereby improving reconstruction accuracy [41].

In reviewing the new developments of mask design, we see interesting directions where the encoder (i.e., mask) has transitioned from static and empirical design to adaptive or learned design. The adaptive encoders introduce reconfigurable optical modulation using programmable devices such as spatial light modulators and digital micromirror arrays. By dynamically adjusting the encoding pattern, these systems can optimize the measurement process for specific imaging tasks or conditions, enabling improved reconstruction quality and broader applicability [37]. The learned encoders treat optical parameters as trainable variables within end-to-end differentiable frameworks, and the imaging hardware and reconstruction algorithms are jointly optimized to maximize task-specific performance [39], [42].

Finally, a recent literature presents a principled framework for the mask design with a goal of maximizing the information captured in measurement, without relying on the downstream reconstruction algorithm [43]. This framework demonstrates that optimal mask design is dictated by the object sparsity and image sensor noise. Extending this principle to include algorithm-specific conditioning represents a promising avenue for broadening its applicability.

### C. Summary of Related Works

In summary, the development of mask-based imaging has been driven by advancements in both optical encoding hardware and computational reconstruction. On the hardware side, the evolution from traditional static encoders to adaptive and learned encoders has enabled more flexible and optimized measurement processes. On the computational side, the shift from classical iterative inversion methods to learned optimization techniques, such as unrolled networks and neural field, has significantly enhanced the quality of reconstructed images. The integration of these advancements has expanded the applicability of mask-based imaging systems across various domains, including biomedical imaging, remote sensing, and industrial inspection, while also opening new avenues for future research and development in this field. Representative optical encoders and reconstruction paradigms are summarized in Tables I and II.

## III. Discussions and Perspectives

### A. Impact

From a technical perspective, DeepInMiniscope overcomes fundamental bottlenecks in computational imaging by dramatically enhancing reconstruction speed, reducing computational complexity, and enabling scalable volumetric imaging over a large FOV. Methodologically, the transition from a monolithic global inversion to a local PSF model and localized reconstruction framework represents a paradigm shift toward spatially structured, parallelizable imaging. This strategic decomposition ensures computational tractability by transforming a globally coupled inverse problem into distributed, independent local processing units. Crucially, this approach ensures that the required GPU memory remains constant even as the total FOV or volume increases; by partitioning the data into smaller, manageable local FOVs, the system can process large-scale volumes sequentially without requiring additional hardware resources.

From a system-level perspective, the architecture of DeepInMiniscope is optimized for real-world deployment. Its compact form factor makes it uniquely suited for miniaturized and wearable applications where physical constraints are critical. Furthermore, the framework is highly extensible. The dimensions of the microlens array and image sensor can be scaled to image larger FOVs, while the underlying ADMM-based reconstruction network can be generalized to a broad class of inverse problems beyond optical microscopy.

This work marks the first demonstration of a mask-based integrated miniscope capable of in vivo imaging of neuronal activity in the awake mouse brain with near-cellular resolution, at depths reaching hundreds of micrometers below the cortical surface. DeepInMiniscope establishes a key step towards large-FOV 3D imaging of neural population dynamics while the animal engages in naturalistic behaviors.

### B. Limitation

Despite the demonstrated advancements of DeepInMiniscope, several constraints remain to be addressed. From the perspective of modeling and reconstruction algorithms, the current framework focuses primarily on spatial recovery and does not yet explicitly incorporate spatiotemporal correlations. By treating each frame as an independent entity, the system may not fully exploit the temporal redundancy typical in dynamic biological processes. From an optical standpoint, light scattering in thick biological tissue remains a fundamental challenge. As imaging depth increases, scattering-induced crosstalk degrades signal integrity, and could limit the system’s effectiveness for deep-brain applications. Furthermore, the samples should generally be sparse to avoid excessive background. From a hardware perspective, the physical footprint of the device is currently constrained by the dimensions of the printed circuit board (PCB), which is ~30 × 30 mm to host the large format sensor (~7.5 × 5.6 mm active region). This current form factor imposes mechanical constraints on the animal when the device is head-mounted, which could impact its natural range of motion and behavioral state.

### C. Future Vision

We envision the development of joint spatiotemporal reconstruction models for DeepInMiniscope that iteratively couple spatial inversion with temporal dynamics in functional recording to improve the fidelity of the extracted signal. Another frontier involves incorporating scattering-aware forward models and learned priors specifically designed for de-scattering to extend imaging depths. The design of the mask can take into account the sparsity of the sample. Finally, the on-going progress in integrated electronics and advanced optical packaging will drive further miniaturization and mechanical robustness of the device. We envision that the next generation of mask-based integrated miniscope will continue to push the boundaries of information encoding and 3D reconstruction in miniaturized, wearable systems.

## IV. Conclusion

DeepInMiniscope represents a breakthrough in mask-based integrate miniaturized microscope. It offers a highly scalable reconstruction framework and the first demonstration of high-resolution in vivo brain imaging in this class of device. The success of this computational imaging scheme underscores the power of joint designing optical hardware and reconstruction algorithms. Such a framework enables real-time, large-scale, and adaptive imaging of complex biological systems.

Finally, as a research highlight, this paper focuses primarily on DeepInMiniscope [23], while also providing a concise overview of recent advancements in mask-based imaging. For a comprehensive discussion of mask-based imaging systems, readers are referred to recent review articles in this field, such as [56], [57], [58].

## Figures and Tables

**TABLE I:** Representative Optical Encoding Strategies

Encoding strategy	Representative works
Coded apertures	Fenimore et al. [44], Asif et al. [13], Nakamura et al. [41]
Microlens array	Adelson et al. [45], Kuo et al. [16], Xue et al. [17], Tian et al. [18], [19], Xue et al. [20], Yang et al. [24], Tian et al. [23]
Phase engineering and Diffuser-based encoding	Antipa et al. [14], Boominathan et al. [15], Adams et al. [21]
Programmable optics	A. Zomet et al. [46], Wu et al. [47], Hua et al. [48], Zhang et al. [37]
Metasurface	Khorasaninejad et al. [49], Fan et al. [50], Hu et al. [40], Long et al. [42]
Learned optical design	Sitzmann et al. [51], Baek et al. [52], Khan et al. [53], Zhang et al. [39]

**TABLE II:** Representative Reconstruction Algorithms

Type of reconstruction algorithms	Representative works
Single step optimization	Asif et al. [13]
Model-based iterative optimization	Antipa et al. [14], Xue et al. [17], Nakamura et al. [41],
Learned unrolling	Monakhova et al. [27], Kingshott et al. [28], Tian et al. [23], [29]
Fourier domain learning	Deb et al. [54], Tian et al. [19], [23], Yang et al. [24]
Neural field reconstruction	Zhang et al. [35], Chien et al. [36]
View synthesis and scene representation	Wang et al. [55], Xue et al. [20]

## References

[1] Flusberg BA , “High-speed, miniaturized fluorescence microscopy in freely moving mice,” Nature Methods, vol. 5, no. 11, pp. 935–938, Nov. 2008, doi: 10.1038/nmeth.1256. 18836457 PMC2828344

[2] Ghosh KK , “Miniaturized integration of a fluorescence microscope,” Nature Methods, vol. 8, no. 10, pp. 871–U147, Oct. 2011, doi: 10.1038/Nmeth.1694. 21909102 PMC3810311

[3] Ziv Y , “Long-term dynamics of CA1 hippocampal place codes,” Nature Neurosci., vol. 16, no. 3, pp. 264–266, Mar. 2013, doi: 10.1038/nn.3329. 23396101 PMC3784308

[4] Cai DJ , “A shared neural ensemble links distinct contextual memories encoded close in time,” Nature, vol. 534, no. 7605, pp. 115–118, Jun. 2016, doi: 10.1038/nature17955. 27251287 PMC5063500

[5] Aharoni D and Hoogland TM, “Circuit investigations with open-source miniaturized microscopes: Past, present and future,” Front Cell Neurosci., vol. 13, Apr. 2019, Art. no. 141, doi: 10.3389/fncel.2019.00141. 31024265 PMC6461004

[6] de Groot A , “NINscope, a versatile miniscope for multi-region circuit investigations,” Elife, vol. 9, Jan. 2020, Art. no. e49987, doi: 10.7554/eLife.49987. 31934857 PMC6989121

[7] Rynes ML , “Miniaturized head-mounted microscope for whole-cortex mesoscale imaging in freely behaving mice,” Nature Methods, vol. 18, no. 4, pp. 417–425, Apr. 2021, doi: 10.1038/s41592-021-01104-8. 33820987 PMC8034419

[8] Guo C , “Miniscope-LFOV: A large-field-of-view, single-cell-resolution, miniature microscope for wired and wire-free imaging of neural dynamics in freely behaving animals,” Sci. Adv, vol. 9, no. 16, Apr. 2023, Art. no. eadg3918, doi: 10.1126/sciadv.adg3918. 37083539 PMC10121160

[9] Scherrer JR, Lynch GF, Zhang JJ, and Fee MS, “An optical design enabling lightweight and large field-of-view head-mounted microscopes,” Nature Methods, vol. 20, no. 4, pp. 546–549, Apr. 2023, doi: 10.1038/s41592-023-01806-1. 36928075

[10] Mait JN, Euliss GW, and Athale RA, “Computational imaging,” Adv. Opt. Photon, vol. 10, no. 2, pp. 409–483, Jun. 2018, doi: 10.1364/Aop.10.000409.

[11] Broxton M , “Wave optics theory and 3-D deconvolution for the light field microscope,” Opt. Exp, vol. 21, no. 21, pp. 25418–25439, Oct. 2013, doi: 10.1364/Oe.21.025418. PMC3867103 24150383

[12] Guo C, Liu W, Hua X, Li H, and Jia S, “Fourier light-field microscopy,” Opt. Exp, vol. 27, no. 18, pp. 25573–25594, Sep. 2019, doi: 10.1364/OE.27.025573. PMC6825611 31510428

[13] Asif MS, Ayremlou A, Sankaranarayanan A, Veeraraghavan A, and Baraniuk RG, “FlatCam: Thin, lensless cameras using coded aperture and computation,” IEEE Trans. Comput. Imag, vol. 3, no. 3, pp. 384–397, Sep. 2017, doi: 10.1109/Tci.2016.2593662.

[14] Antipa N , “DiffuserCam: Lensless single-exposure 3D imaging,” Optica, vol. 5, no. 1, pp. 1–9, Jan. 2018, doi: 10.1364/Optica.5.000001.

[15] Boominathan V, Adams JK, Robinson JT, and Veeraraghavan A, “PhlatCam: Designed phase-mask based thin lensless camera,” IEEE Trans. Pattern Anal. Mach. Intell, vol. 42, no. 7, pp. 1618–1629, Jul. 2020, doi: 10.1109/Tpami.2020.2987489. 32324539 PMC7439257

[16] Kuo G, Linda Liu F, Grossrubatscher I, Ng R, and Waller L, “On-chip fluorescence microscopy with a random microlens diffuser,” Opt. Exp, vol. 28, no. 6, pp. 8384–8399, Mar. 2020, doi: 10.1364/OE.382055. 32225465

[17] Xue Y, Davison IG, Boas DA, and Tian L, “Single-shot 3D wide-field fluorescence imaging with a computational miniature mesoscope,” Sci. Adv, vol. 6, no. 43, Oct. 2020, Art. no. eabb7508, doi: 10.1126/sciadv.abb7508. 33087364 PMC7577725

[18] Tian F, Hu J, and Yang W, “GEOMScope: Large field-of-view 3D lensless microscopy with low computational complexity,” Laser Photon Rev., vol. 15, no. 8, Aug. 2021, Art. no. 2100072, doi: 10.1002/lpor.202100072. 34539926 PMC8445384

[19] Tian F and Yang W, “Learned lensless 3D camera,” Opt. Exp, vol. 30, no. 19, pp. 34479–34496, Sep. 2022, doi: 10.1364/OE.465933. PMC9576281 36242459

[20] Xue Y, Yang Q, Hu G, Guo K, and Tian L, “Deep-learning-augmented computational miniature mesoscope,” Optica, vol. 9, no. 9, pp. 1009–1021, Sep. 2022, doi: 10.1364/OPTICA.464700. 36506462 PMC9731182

[21] Adams JK , “In vivo lensless microscopy via a phase mask generating diffraction patterns with high-contrast contours,” Nature Biomed. Eng, vol. 6, no. 5, pp. 617–628, May 2022, doi: 10.1038/s41551-022-00851-z. 35256759 PMC9142365

[22] Wu J, Chen Y, Veeraraghavan A, Seidemann E, and Robinson JT, “Mesoscopic calcium imaging in a head-unrestrained male non-human primate using a lensless microscope,” Nature Commun., vol. 15, no. 1, Feb. 2024, Art. no. 1271, doi: 10.1038/s41467-024-45417-6. 38341403 PMC10858944

[23] Tian F, Mattison B, and Yang W, “DeepInMiniscope: Deep learning-powered physics-informed integrated miniscope,” Sci. Adv, vol. 11, no. 37, Sep. 2025, Art. no. eadr6687, doi: 10.1126/sciadv.adr6687. 40938981 PMC12429034

[24] Yang Q, Guo R, Hu G, Xue Y, Li Y, and Tian L, “Wide-field, high-resolution reconstruction in computational multi-aperture miniscope using a Fourier neural network,” Optica, vol. 11, no. 6, pp. 860–871, Jun. 2024, doi: 10.1364/OPTICA.523636. 39895923 PMC11784641

[25] Xiong Z, He W, Chen Y, Xu Y, Wang W, and Fu Y, “Lensless imaging through the fourier-ADMM network with a cost-effective and easily fabricated phase mask,” Opt. Lett, vol. 50, no. 3, pp. 758–761, Feb. 2025, doi: 10.1364/OL.549538. 39888746

[26] Li YY, Zhang ZX, Tian F, Luna-palacios YY, Rocha-mendoza I, and Yang WJ, “V-shaped PSF for 3D imaging over an extended depth of field in wide-field microscopy,” Opt. Lett, vol. 50, no. 2, pp. 383–386, Jan. 2025, doi: 10.1364/Ol.544552. 39815517

[27] Monakhova K, Yurtsever J, Kuo G, Antipa N, Yanny K, and Waller L, “Learned reconstructions for practical mask-based lensless imaging,” Opt. Exp, vol. 27, no. 20, pp. 28075–28090, Sep. 2019, doi: 10.1364/Oe.27.028075. 31684566

[28] Kingshott O, Antipa N, Bostan E, and Aksit K, “Unrolled primal-dual networks for lensless cameras,” Opt. Exp, vol. 30, no. 26, pp. 46324–46335, Dec. 2022, doi: 10.1364/OE.475521. 36558589

[29] Tian F and Yang W, “Unrolled Richardson-Lucy deconvolution network with partially connected layers in computational microscopy,” Opt. Exp, vol. 34, no. 10, pp. 18676–18689, May 2026, doi: 10.1364/OE.588318. 42271986

[30] Yanny K, Monakhova K, Shuai RW, and Waller L, “Deep learning for fast spatially varying deconvolution,” Optica, vol. 9, no. 1, pp. 96–99, Jan. 2022, doi: 10.1364/Optica.442438.

[31] Howard S, Norreys P, and Döpp A, “CoordGate: Efficiently computing spatially-varying convolutions in convolutional neural networks,” in Proc. 34th Brit. Mach. Vis. Conf, 2023. [Online]. Available: https://proceedings.bmvc2023.org/744/

[32] Yang Q, Chen Z, Zhang J, Guo R, Hu G, and Tian L, “Coordinate-conditioned deconvolution for scalable spatially varying high-throughput imaging,” 2026, arXiv:2602.01065. 10.1109/tci.2026.3715066 PMC13560750 42725122

[33] Mildenhall B, Srinivasan PP, Tancik M, Barron JT, Ramamoorthi R, and Ng R, “NeRF: Representing scenes as neural radiance fields for view synthesis,” Commun. Acm, vol. 65, no. 1, pp. 99–106, Jan. 2022.

[34] Xie Y , “Neural fields in visual computing and beyond,” Comput. Graph. Forum, vol. 41, no. 2, pp. 641–676, 2022, doi: 10.1111/cgf.14505.

[35] Zhang OM , “Single-shot volumetric fluorescence imaging with neural fields,” Adv. Photon, vol. 7, no. 2, Mar. 2025, Art. no. 026001, doi: 10.1117/1.Ap.7.2.026001.

[36] Chien T, Cao R, Liu FL, Kabuli LA, and Waller L, “Space-time reconstruction for lensless imaging using implicit neural representations,” Opt. Exp, vol. 32, no. 20, pp. 35725–35732, Sep. 2024, doi: 10.1364/OE.530480. 40514926

[37] Zhang X , “Lensless imaging with a programmable Fresnel zone aperture,” Sci. Adv, vol. 11, no. 12, Mar. 2025, Art. no. eadt3909, doi: 10.1126/sciadv.adt3909. 40117355 PMC11927639

[38] Yu Q, Huang M, Wang Y, Wan W, and Liu Q, “High-quality FZA lensless imaging via joint generation of original-twin images,” Photon. Res, vol. 14, no. 5, pp. 1749–1761, May 2026, doi: 10.1364/PRJ.584406.

[39] Zhang Y , “A miniaturized mesoscope for the large-scale single-neuron-resolved imaging of neuronal activity in freely behaving mice,” Nature Biomed. Eng, vol. 8, no. 6, pp. 754–774, Jun. 2024, doi:10.1038/s41551-024-01226-2. 38902522

[40] Hu JJ and Yang WJ, “Metalens array miniaturized microscope for large-field-of-view imaging,” Opt. Commun, vol. 555, Mar. 2024, Art. no. 130231, doi: 10.1016/j.optcom.2023.130231.

[41] Nakamura T, Kato R, Iwata K, Makihara Y, and Yagi Y, “Multilayer lensless camera for improving the condition number,” Appl. Opt, vol. 63, no. 28, pp. G9–G17, Oct. 2024, doi: 10.1364/Ao.521126.

[42] Long Z, Zeng Y, and Jin X, “End-to-end design of the metalens array imaging system with extended depth of field,” Opt. Exp, vol. 33, no. 13, pp. 26869–26886, Jun. 2025, doi: 10.1364/OE.560354. 40798243

[43] Kabuli LA, Pinkard H, Markley E, Hung CS, and Waller L, “Designing lensless imaging systems to maximize information capture,” Optica, vol. 13, no. 2, pp. 227–235, Feb. 2026, doi: 10.1364/OPTICA.570334.

[44] Fenimore EE and Cannon TM, “Coded aperture imaging with uniformly redundant arrays,” Appl. Opt, vol. 17, no. 3, pp. 337–347, 1978, doi: 10.1364/Ao.17.000337. 20174412

[45] Adelson EH and Wang JYA, “Single lens stereo with a plenoptic camera,” IEEE Trans. Pattern Anal. Mach. Intell, vol. 14, no. 2, pp. 99–106, Feb. 1992, doi: 10.1109/34.121783.

[46] Zomet A and Nayar SK, “Lensless imaging with a controllable aperture,” in Proc. IEEE Comput. Soc. Conf. Comput. Vis. Pattern Recognit, Jun. 2006, vol. 1, pp. 339–346, doi: 10.1109/CVPR.2006.175.

[47] Wu Y, Sharma MK, and Veeraraghavan A, “WISH: Wavefront imaging sensor with high resolution,” Light, Sci. Appl, vol. 8, no. 1, May 2019, Art. no. 44, doi: 10.1038/s41377-019-0154-x. 31069074 PMC6491653

[48] Hua Y, Nakamura S, Asif MS, and Sankaranarayanan AC, “Sweep-Cam - Depth-aware lensless imaging using programmable masks,” IEEE Trans. Pattern Anal, vol. 42, no. 7, pp. 1606–1617, Jul. 2020, doi: 10.1109/Tpami.2020.2986784. 32305898

[49] Khorasaninejad M and Capasso F, “Metalenses: Versatile multifunctional photonic components,” Science, vol. 358, no. 6367, Dec. 2017, Art. no. eaam8100, doi: 10.1126/science.aam8100. 28982796

[50] Fan Q , “Trilobite-inspired neural nanophotonic light-field camera with extreme depth-of-field,” Nature Commun., vol. 13, no. 1, Apr. 2022, Art. no. 2130, doi: 10.1038/s41467-022-29568-y. 35440101 PMC9019092

[51] Sitzmann V , “End-to-end optimization of optics and image processing for achromatic extended depth of field and super-resolution imaging,” ACM Trans. Graph, vol. 37, no. 4, Jul. 2018, Art. no. 114, doi: 10.1145/3197517.3201333.

[52] Baek S-H , “Single-shot hyperspectral-depth imaging with learned diffractive optics,” in Proc. IEEE/CVF Int. Conf. Comput. Vis, Oct. 2021, pp. 2631–2640, doi: 10.1109/ICCV48922.2021.00265.

[53] Khan SS, Boominathan V, Veeraraghavan A, and Mitra K, “Designing optics and algorithm for ultra-thin, high-speed lensless cameras,” in Proc. IEEE Int. Conf. Multimedia Expo, Jul. 2023, pp. 1583–1588, doi: 10.1109/ICME55011.2023.00273.

[54] Deb D , “FourierNets enable the design of highly non-local optical encoders for computational imaging,” in Proc. 36th Int. Conf. Neural Inf. Process. Syst, 2022, Art. no. 1829.

[55] Wang Z , “Real-time volumetric reconstruction of biological dynamics with light-field microscopy and deep learning,” Nature Methods, vol. 18, no. 5, pp. 551–556, May 2021, doi: 10.1038/s41592-021-01058-x. 33574612 PMC8107123

[56] Boominathan V, Robinson JT, Waller L, and Veeraraghavan A, “Recent advances in lensless imaging,” Optica, vol. 9, no. 1, pp. 1–16, Jan. 2022, doi: 10.1364/Optica.431361. 36338918 PMC9634619

[57] Li S , “Lensless camera: Unraveling the breakthroughs and prospects,” Fundam. Res, vol. 5, pp. 1725–1736, 2024, doi: 10.1016/j.fmre.2024.03.019. 40777808 PMC12327861

[58] Wang F, Czarske JW, and Situ G, “Deep learning for computational imaging: From data-driven to physics-enhanced approaches,” Adv. Photon, vol. 7, no. 5, Sep. 2025, Art. no. 054002, doi: 10.1117/1.Ap.7.5.054002.

